# New Appliances and Things Medical

**Published:** 1896-03-28

**Authors:** 


### NEW APPLIANCES AND THINGS MEDICAL.

[We shall be glad to receive, at our Office, 428, Strand, London, W.C., from the manufacturers, specimens of all new preparations and appliances, which may be brought out from time to time.]

### ALLAN'S CHILD CORDIAL OR CORRECTIVE.

(T. H. Allan, 59, Beck Road, Liverpool.)

We have studied the prescription which Mr. Allan gives of this cordial, and regard it as a combination of carminative drugs likely to prove serviceable in the treatment of some disorders of the nature of wind or indigestion. Chloral or opium finds no place in the prescription with which we have been furnished. It must not be forgotten, however, that medi ine is not the only corrective for these conditions. Injudicious feeding is almost invariably at the root of the evil.

### THE ELLISON BREAST BANDAGE.

W. H. Bailey and Son, 38, Oxford Street, London, W.

We were pleased to see that a most useful little invention has been patented by Nurse Ellison. The want of breast supporters for newly-accouched women has long been experienced. We feel confident that this appliance will prove a great boon, not only to nursing mothers but also to patients who may need poultices or dressings to be kept in place, by a bandage which occasions neither discomfort nor inconvenience. The price is 5s. 6d.

